# Hot Spring Metagenomics

**DOI:** 10.3390/life3020308

**Published:** 2013-04-25

**Authors:** Olalla López-López, María Esperanza Cerdán, María Isabel González-Siso

**Affiliations:** Departamento de Bioloxía Celular e Molecular, Facultade de Ciencias, Universidade da Coruña, 15071 A Coruña, Spain; E-Mails: olopez@udc.es (O.L.-L.); esper.cerdan@udc.es (M.E.C.)

**Keywords:** metagenomics, hot springs, thermophiles

## Abstract

Hot springs have been investigated since the XIX century, but isolation and examination of their thermophilic microbial inhabitants did not start until the 1950s. Many thermophilic microorganisms and their viruses have since been discovered, although the real complexity of thermal communities was envisaged when research based on PCR amplification of the 16S rRNA genes arose. Thereafter, the possibility of cloning and sequencing the total environmental DNA, defined as metagenome, and the study of the genes rescued in the metagenomic libraries and assemblies made it possible to gain a more comprehensive understanding of microbial communities—their diversity, structure, the interactions existing between their components, and the factors shaping the nature of these communities. In the last decade, hot springs have been a source of thermophilic enzymes of industrial interest, encouraging further study of the poorly understood diversity of microbial life in these habitats.

## 1. Introduction

Currently there is a great interest in hot springs, which are the natural habitat of thermophilic and hyperthermophilic microorganisms with optimal growth temperatures of >55 °C and >80 °C, respectively. Enzymes obtained from them have been proved to be extremely valuable as biocatalysts for industrial and biotechnological purposes. A paradigm is Taq polymerase from *Thermus aquaticus* that led to the development of the polymerase chain reaction (PCR) technique [1].

The initial studies on hot springs focused only on their physicochemical properties and geological features, and it was not until the mid-XX century that the study of the microbiology of these ecosystems began [2]. The temperature in hot springs is usually over the limit of eukaryotic life (near to 60 °C), which limits the microbial life to Bacteria and Archaea (and their viruses). The earliest microbiological work was based on the isolation and identification of thermophilic microbial strains. 

16S rRNA-based studies subsequently revealed that microbial diversity was much broader than suggested by culture-dependent techniques. In combination with the construction of metagenomic libraries, research on total environmental DNA produced a vast amount of information, providing detailed pictures of the microbial communities present in diverse thermal environments. Each hot spring differs from others in temperature, chemical composition and its gradients of temperature or light. Hot springs comprise several habitats, such as thermal fluids, microbial mats and sediments. This diversity of habitats provides a vast number of sites to sample, all with potential interest for metagenomic analysis. The increasing number of reports makes it easier to understand how physicochemical conditions and biological interactions have shaped these microbial communities within their specific environments. In this review, we will illustrate with several examples the usefulness of metagenomic techniques in expanding our knowledge about microbial communities in hot springs.

## 2. Bacteria and Archaea

The first studies on thermophilic microorganisms from hot springs were focused on isolation and characterization of thermophilic strains using culture-dependent approaches [2]. However, ~99% of the microorganisms within a particular environment proved uncultivable [3], although culture methods were improved to try to overcome the requirements of some of these reluctant strains that showed stringent growth conditions or inter-dependent microbial consortia to live [4]. The estimated number of microbial species that might be detected by further development of this methodology is still difficult to predict. 

New molecular methodology gives support to the study of the whole biological diversity through a metagenomic approach that might help to characterize all the microorganisms living in an environment. The total present DNA, called “metagenome”, is extracted and purified from environmental samples, which includes microorganisms that cannot be cultured. Molecular phylogenetic methods, based on the comparison of the sequences from the 16S rRNA genes, have revealed the hidden diversity of hot springs using the metagenomic approach. They have been extensively used to describe microbial communities in hot springs and identify novel thermophilic microorganisms since the mid-1990s. Early microbial diversity analysis combined PCR amplification of the 16S rRNA genes and their pattern analysis on denaturing gradient gel electrophoresis (DGGE). This provided the first insight into the true diversity of these environments, but only DNA sequencing of the amplified targets allowed that new microorganisms, most of them non-cultivable, could be identified and classified. More recently, the development of next-generation sequencing technologies allows metagenomic libraries to be rapidly constructed and sequenced. Therefore the sequences of the 16S rRNA genes or other relevant genes can easily be analyzed, providing a more comprehensive view of microbial diversity.

### 2.1. PCR Approach

The first PCR-based studies of 16S rRNA genes, carried out on organisms from the hot springs of Yellowstone National Park (YNP), revealed an unexpectedly high diversity in contrast with the estimate from culture-dependent studies. This finding led to a revolution in the understanding of phylogenetic relations between Archaea and Bacteria [5,6]. Since then, the same strategy has been used to analyze community profiles from hot springs all over the world. Phylogenetic diversity was greatly expanded with the finding of novel phylotypes, especially in the predominant phylogenetic groups from these hot springs, *i.e.*, the archaeal phylum *Crenarchaeota* and the bacterial division *Aquaficales* [6,7], and even to the discovery of a new archaeal phylum classified as the *Korarchaeota* [6]. However, the also new archaeal phylum *Nanoarchaeota* remained undetectable by conventional PCR-based studies until it was discovered using non-traditional culturing techniques. It is represented by *N. equitants,* a nanosized hypertermophilic symbiont from a submarine hot vent, whose rRNA gene sequence is unique, even in the highly conserved regions used as primer targets for PCR [8]. The newly found *Nanoarchaeota* and *Korarchaeota* are members of the deepest branch-offs of the rRNA phylogenetic tree [9] 

Some approaches went far beyond by using additional biomolecular markers to analyze in parallel the microbial diversity. For instance, given the high abundance of the polysaccharide chitin in marine environments, chitinase genes were analyzed in coastal hot springs, that provide high diversity of valuable new chitinase genes [10]. From a hot spring in Bulgaria, the phylogeny of the archaeal community was analyzed using 16S RNA genes and genes of the glycoside hydrolase-4 family. The good correspondence between both affiliation assignment methods proved the usefulness of these gene encoding metabolic enzymes for phylogenetic studies in heterotrophic archaea [11]. The study also allowed the direct cloning of these genes of industrial interest [11].

Temperature is seen as the main factor that shapes microbial communities in hot springs with different locations [12]. Most hot springs limit the survival of eukaryotic organisms (their limiting temperature for thermophiles reaching 60 °C) and the presence of photosynthetic organisms (70 °C). A particular example is the unique coastal hot springs in Iceland, where the microbial populations are exposed to fluctuations of temperature (and salinity) as a consequence of periodic high tides. The areas suffering the longest hot-temperature periods showed a majority of terrestrial thermophilic bacteria, whereas the areas with the shortest hot-temperature periods showed predominance of moderately thermophilic microorganisms and the presence of mesophilic marine microorganisms and proteobacteria [10].

Although the structure of a bacterial community seems to be mainly determined by temperature, specific populations may be subjected to other factors. Geochemical features of hot springs might act as key determinants in community structure and diversity [7]. There is evidence that composition of an actinobacterial community is influenced by a combination of temperature and pH; this study also reveals the high degree of endemism in this group among different hot springs, even in those that are geographically distant [13].

Emerging pyrosequencing techniques overcome the otherwise limited sampling in library construction based on PCR amplified fragments and Sanger sequencing. Deep sampling achieves a better coverage of the microbial diversity, which is necessary to detect rare and minority microorganisms. Using these powerful techniques, diverse research groups have depicted a general view of thermal communities and have assigned abundance percentages to each identified phylum. The data confirm the predominance of the previously defined groups, but also show novel phylotypes related to specific features related to particular hot springs. The hot springs in Africa, which have been sampled hitherto, are dominated by phylotypes belonging to the *Proteobacteria* [14]. Groundwater in a thermal field in Russia shows that Archaea is dominated by a novel division in the phylum *Euryarchaeota* related to the order *Thermoplasmatales* (39% of all archaea) and by another abundant group (33% of all archaea) related to MCG1 lineage of the phylum *Crenarchaeota* [15]; both groups are widely spread in hot springs all over the world [15]. However, bacteria are dominated by thermoacidophilic methanotrophs and sulfur-oxidizing microorganisms that use inorganic substrates of volcanic origin [15]. The analysis of the taxonomic and metabolic features of the microbial community of a Colombian acidic hot spring showed that only a small proportion of the metagenomic sequences had matches against databases, possibly due to a high proportion of novel taxa; some groups potentially involved in the nitrogen and sulfur cycling in this environment have been described [16].

Temperature, as a shaping agent in a bacterial microbial mat of effluent channels from two hot springs in YNP, was analyzed by bar-coded pyrosequencing [12]. This technique entails the introduction of different codes in the primers used with the different samples so as to assign a sample origin to each sequence retrieved after pyrosequencing. Samples along the temperature gradient showed a loss of diversity and richness with increasing temperatures, probably as a consequence of the effect on primary producers affected by temperatures near the maximum that permits photosynthesis. Distribution of Cyanobacteria and *Chloroflexi* along the thermal gradient was the subject of special investigation. The results showed a general tendency to an alternative presence of the two groups in the abundance peak, which suggests that competition exists for physical space and/or limited nutrients. This explanation was proposed as more reliable than the longstanding hypothesis that co-adapted lineages of these bacteria maintain tightly co-occurring distributions along the gradient as a result of a producer-consumer relationship. In conclusion, temperature is revealed as the main factor in shaping bacterial microbial communities in hot springs of YNP, despite different taxon composition supported by specific solutes and physicochemical properties of water [12].

Differences in the importance of geographical distance and divergence of community structure were also found. A number of microbial species may be ubiquitous in hot springs. Some studies show the relevance of certain taxa distributed in hot springs world-wide, such as pJP89-related organisms and uncultured groups of the order *Thermoplasmatales* [15]. Nevertheless, other taxa seem to be endemic in certain regions [13,17]. The importance of geographic distance is controversial, but may be a significant factor that influences microbial communities according to some studies focused on actinobacteria and cyanobacteria populations [13,18], although this proved not to be significant in other studies [12]. The differences of diversity observed among bacterial populations cannot be attributable to the distance between hot springs, but to temperature variations in the hot springs [12].

### 2.2. Metagenomic Libraries

Metagenomic libraries yield comprehensive community profiles, and additional information on the lifestyles of the microorganisms present in environmental samples allow entire or partial community metabolic fluxes to be reconstructed. This approach leads to differences in community composition compared with PCR-based methods [19], and each one has advantages and disadvantages. PCR amplification of 16S rRNA may produce a bias due to the unequal amplification of species and chimeric sequences, whereas metagenomics may fail to detect rare species in a community. Uncultured bacteria can be detected by traditional studies of microbial diversity by PCR of 16S rRNA, but assignment to a metabolic type is difficult to achieve when there are not related cultivable microorganisms [7]. 

The identification of novel microbial species or phylotypes by analysis of 16S rRNA genes is a task not as simple to fulfil as is the species demarcation in animal and plants. There is a comprehensive review about this issue [20] that questions the reliability of selecting a cut-off of 97-98% for similarity in 16S rRNA sequences as a good criterion to discriminate new microbial species from closely related microorganisms. Those demarcated by the 97-98% cut-off might include different species-like ecotypes, which could potentially lead to an underestimate of the real microbial diversity in an environment. Tindall *et al.* [21] provide recommendations for analysis of the 16S rRNA genes in the field of prokaryote taxonomy. They indicate that when 16S rRNA gene similarity values are over 97% (over full pairwise comparisons) it is necessary to use other methods to discriminate new species, such as DNA-DNA hybridization or analysis of gene sequences with a greater resolution; these methods must also be correlated with phenotype characterization. At values above 95% of 16S rRNA gene similarity (over full pairwise comparisons), taxa should also be tested by other methods in order to establish whether separate genera are present.

Using metagenomics, genomes can be assembled and thereafter 16S rRNA can be analyzed along with other relevant genes, thereby more accurately demarcating new species. One example carefully analyzed is the cyanobacteria, *Synechococcus*, an inhabitant of microbial mats in hot springs along thermal gradients where ecologically different subpopulations have been found by exhaustive metagenomic analysis [22]. These subpopulations differ in certain metabolic capabilities and their genomes lack a conserved large-scale genomic order. This study highlights how useful metagenomics can be in understanding the heterogeneity of species in a natural microbial population. Metagenomic libraries are potent tools to identify and describe novel uncultured taxa, by assembly of their genomes or simply by exploring the metagenome, searching for functional genes to infer metabolic features and phylogenetic markers. This is a difficult task, given that often these uncultured members are minor components in a highly diverse community; besides, metagenomic information extracted from sequenced libraries can be partial and fragmented. Nevertheless, several new bacteria have been described in metagenomic libraries of microbial mats of hot springs, apart from the dominant populations of microbial mats (*Synechococcus*, *Chloroflexi* and *Roseiflexus* spp.-related strains), indicating that the microbial community is more phylogenetically and physiologically diverse than the studies of their 16S rRNA genes might indicate. For instance, discovery of a new member of anoxygenic chlorophototrops within the phylum *Chloroflexi*—where chlorotrophy was thought to be restricted to the class *Chloroflexi* [23], or the first phototrophic member of the phylum *Acidobacteria*, which was detected by BLAST search in metagenomic libraries of sequences from components of the type 1 reaction centre of chlorophototrophs [24]. The assignment of novel metabolic capabilities to these newly discovery taxa groups were only possible by analysis of available metagenomic information. In this way, an uncultured member of the phylum *Chlorobi*, provisionally named *Candidatus Thermochlorobacter aerophilum*, was found in the microbial mats of one alkaline siliceous hot springs at the YNP and was investigated through metagenomic and metatranscriptomic approaches. It was proposed to be an aerobic photoheterotroph that cannot oxidize sulfur compounds, cannot fix N_2_, and does not fix CO_2_ autotrophically. Metagenomic analyses suggest that it depends on other organisms in the mat, which provide fixed carbon and nitrogen, several amino acids, and other important nutrients [25]. Also, metagenomics of hydrothermal vents revealed the potential importance of H_2_ as a key energy source in the deep ocean [26].

Recently, the partial assemble of the genome from a single cell became possible. In this way, the genome from one uncultured bacteria isolated from sediments of a hot spring in Oklahoma, which belongs to the world widely distributed candidate division OP11, provided important information about its lifestyle (metabolic capabilities, secretory pathways, cell wall structure, and defence mechanisms). These results should be taken with caution, since the genome of a single cell cannot be representative of the entire lineage [27].

There are many examples of metagenomic libraries used to investigate the diversity of microbial communities of hot springs in more complex studies and most of them were carried out in YNP: For instance, a detailed picture of community structure and ecology of a microbial mat sample from Mushroom hot spring was provided by comprehensive metagenomic studies of environmental DNA and RNA, which analyzed several phylogenetic and functional relationships between genes [23,28]. Metatranscriptome analysis at different time-points: during light-to-dark and during dark-to-light transitions showed different temporal pattern behavior in the different phototrophic populations [28]. 

One of the new issues addressed by metagenomics is an approach to understand the functional role of mobile genetic elements, e.g. insertion sequences (IS) [29]. These have the ability to produce rearrangements in the genome, making them a powerful force in genome evolution. Metagenomics provides a powerful method to gain an insight into the IS genome content and its location in a natural population, and to learn about the mechanisms of IS accumulation and survival against mutational forces. Clone ends usually show putative affiliation to different reference genomes in some metagenomic libraries. This suggests the existence of previous gene exchanges, even between distant lineages, many of them related with CRISPR (Clusters of Regularly Interspaced Short Palindromic Repeats)—associated proteins that have phage-host functions [23,30]. The thermophilic cyanobacterium, *Synechococcus,* shows many of such rearrangements in its genome by comparison among different strains, but which are dominant in a specific habitat. The abundance of IS in reference genomes of strains A and B’ did not differ from natural communities, and provides evidence of recent lateral gene transfers between ecotype-like species. Furthermore, metagenomics provide a snapshot of the population, and allows the detection of deleterious mutations caused by IS that have not yet been selected against [29]. Comparative metagenomics of microbial communities inhabiting deep-sea hydrothermal vent chimneys showed a high proportion of transposases in the metagenomes, implying that horizontal gene transfer may be a common occurrence in the deep-sea vent chimney biosphere [31].

As well as with 16S rRNA based strategies, there are comprehensive studies where both temperature and geochemical composition were seen as factors that shape microbial communities from a metagenomic approach. A metagenomic study based on the Bison Pool hot spring (YNP) microorganism inhabitants in the effluent channel revealed a continuum of different microbial communities along a gradient of temperature and geochemical conditions, with different metabolic capabilities and lifestyles in accordance with these changing environmental conditions. Dominant taxa were identified in each sample location, and it was also possible to differentiate between specialist and generalist components in the microbial community [32]. 

## 3. Viruses

Viruses are the most abundant biological entities in every ecosystem, even among hot springs, although several estimates indicate a density lower than in other mesophilic aquatic systems [33]. Studies of viruses in this branch of environmental metagenomics do not only pursue the identification and classification of new types but also aim for a better understanding of the role of viruses in nature, and whether (and if so, how) they interact with other elements of the ecological systems. They are probably the only predators in these communities, and may be involved in the control of host mortality and the carbon cycle [33].

In the beginning, the only methodology available to study a virus was to culture its host, implying that studies were biased for lytic types in bacteriophages, which hindered further progress in this field. Metagenomic studies on viruses from thermal environments overcame this limitation by showing the enormous diversity and abundance of viruses in these ecosystems. For example, the consensus genome sequence of a novel GC-rich archaeal rudivirus recovered by iterative *de novo* read mapping and assembly from a hot spring metagenome was recently reported [34]. Nevertheless, enriched cultures continue to be used to identify new viruses with success. A bioreactor inoculated with a sample from a hot spring in YNP was maintained for two years at 85 °C and pH 6 and two new viruses were discovered by this approach [35].

Metagenomics is providing a vast amount of information about viruses in hot springs, but correct handling and understanding of new data remains in progress. Population dynamics are also being studied, but the data are poorly understood [36], the biggest problem being the correct virus classification. Viruses have been classified according to their host range and morphology, owing to the lack of universal genomic signatures like the 16S rRNA genes of prokaryotes. Failure to find homology to sequences coming from new viral metagenomes in GenBank using BLAST alignments made necessary the development of other tools. An alternative method is viral genome signature-based phylogenetic classification, using a database of oligonucleotide frequencies instead of sequence similarity, which gives a more reliable classification of the viral sequences and the assignment of a likely host [37]. 

Another problem is the bias in the studies. Most viruses being investigated are double-stranded DNA due to the methodology of library construction, but advances in this procedure made the study of RNA viruses possible. The first genome segments belonging to a putative positive-strand RNA virus replicating in archaeal hosts were recently isolated from several acidic hot springs of YNP. The virus might be related to the direct ancestor or eukaryotic viruses, whose origin remains unknown [38]. 

The first metagenomic study of viruses in geothermal environments was carried out relatively recently, with double-stranded DNA viruses of the Octopus and Bear Paw hot springs (YNP). The assembly of viral metagenomes indicated a high degree of heterogeneity, with the predominance of a lytic lifestyle. The occurrence of the appropriate machinery for lateral gene transfers and evidence of the replacement of cellular genes by non-orthologous viral genes (*i.e.*, the similarity between several proteins from cell and virus, such as helicases and DNA polymerases) suggested that viruses might play a critical role in the evolution of DNA and its replication mechanisms [33].

In the genomes of Bacteria and Archaea, there are loci called CRISPR that act as a molecular registration of phage attacks on the cell. They contain virus-derived sequences originating from previous viral infections or acquired by lateral transfers, which confer immunity against the phage that also contains one of them in its genome. Therefore, comparative analysis of viral sequences and cellular CRISPR might provide information about the archaeal or bacterial hosts of viruses [30,39]. A microarray assay has been designed to detect and analyze these viral sequences in the environmental samples, using metagenomic sequences from bacteria and archaea inhabiting acidic hot springs as probes. Effectively, it demonstrated its usefulness in detecting new viruses and monitoring changes in a viral population [36]. This method gives similar results to those obtained by the construction of metagenomic libraries, but it is less expensive and time-consuming.

Finally, metagenomic techniques show evidence of homologous recombination between viruses. The presence of capsid protein from RNA viruses in cirvovirus-like DNA suggest an event of gene transfer between unrelated RNA and DNA viruses, but the molecular mechanisms involved remain unclear. Further studies in metagenomic libraries might help to decipher virus evolution [40].

## 4. Novel Thermophilic Enzymes

Thermostable enzymes from thermophilic microorganisms are important biocatalysts for industrial and biotechnological purposes, given that they can work at high temperatures in which mesophilic enzymes would be denatured. There are now many thermophilic enzymes being used for biotechnological and industrial purposes. The classical example is Taq DNA polymerase from *Thermus aquaticus,* purified and isolated from hot springs [1], which made the development of the PCR amplification technique possible. Studies of the biodiversity in hot springs revealed the presence of complex communities containing novel microorganisms, which can be potential sources of novel enzymes with unique features of interest in industrial applications.

Thermophilic enzymes were primarily screened in a culture-based manner, but metagenomics of hot springs facilitate the search of new biocatalysts by functional screening for the desired activity or by shotgun sequencing and the search for the target enzyme in metagenomic libraries.

Sequence-based approaches are biased towards already-known families of enzymes, but some authors prefer this approach to circumvent problems ingetting the correct expression of the foreign gene coding in the desired enzyme in a heterologous host. This was the case with a thermostable Fe-superoxide dismutase that was identified from a partially sequenced metagenomic library using BLAST. The activity of this enzyme that eliminates toxic superoxide radicals has potential use as an additive in the cosmetic industry [41]. More recently, three novel genes conferring lipolytic and one gene conferring proteolytic activity were identified by mining a thermal spring volcanic metagenome. These genes were cloned into expression vectors and the recombinant proteins characterized showing special features [42].

By functional screening, entirely new classes of enzymes can be found, but the success of this approach requires the target protein to be compatible with transcription and translation machinery of the host. Although only 40% of the foreign proteins expressed in *E. coli* appear to be successfully expressed [43], several lipolytic enzymes, with potential industrial applications, have been isolated by functional screening from thermal springs in Thailand [44]. A curious case was a lipase isolated from soil in a hot spring area in India, which was thermo-labile but of thermophilic origin [45]. Thermostability of this protein was successfully improved by protein engineering, the mutated form being 144-fold more stable at 60 °C than the native enzyme [46].

Many DNA polymerases from thermophilic microorganisms have been exploited for biotechnological purposes since the discovery of the Taq polymerase. But the number of enzymes utilized for biotechnological purposes is strikingly low compared to the tremendous amount of diversity that exists in viral genes. A thermostable DNA polymerase of viral origin was isolated from a viral metagenomic library of Octopus hot spring in YNP [33] by identification of potential polymerase genes using BLAST alignment prior to functional screening. The most thermostable enzyme possesses reverse transcriptase and DNA polymerase activities. It has been modified to eliminate exonuclease activity, and the engineered enzyme has turned out to be a viable alternative to the traditional 2-enzyme systems employed in RT-PCR, with both higher specificity and sensitivity [47].

## 5. Conclusions

Less diversity and richness in hot springs than in other aquatic environments have been reported [12,33,48]. Analysis of thermal communities from all over the world has found the recurrent presence of certain groups, although in some hot springs there are specific strains that contribute significantly to the composition of the microbial community, and whose presence is correlated to the geochemical properties of hot springs. Apart from microbial community profiles, metagenomics assesses the effect of physicochemical conditions in community diversity from hot springs; although temperature seems to be the major factor, geochemical compositions and geographical distances are significant in some cases. 

Discoveries in thermal environments have increased the knowledge about the evolution not only of bacteria and archaea, but also of viruses. As far as metagenomic studies are to be expanded, a more complete phylogeny of these groups could be drawn.

Hot springs are also a vast source of new and diverse thermophilic enzymes, many with potential uses in industry. Metagenomics is a powerful tool to identify yet unknown enzymes and provide industry with more cost-effective biocatalysts for specific purposes.

The reports summarized in this review and others not covered for brevity, but equally valuable, demonstrate the unequalled potential of metagenomics in the study of hot springs as well as the importance of continuing research in this field. We might expect that the findings already made probably represent only the tip of the iceberg.
